# Circulatory white spot syndrome virus in South-West region of Bangladesh from 2014 to 2017: molecular characterization and genetic variation

**DOI:** 10.1186/s13568-018-0553-z

**Published:** 2018-02-20

**Authors:** Mohammad Anwar Siddique, Md. Inja-Mamun Haque, Santonu Kumar Sanyal, Anwar Hossain, Shuvro Prokash Nandi, A. S. M. Rubayet Ul Alam, Munawar Sultana, Mahmud Hasan, M. Anwar Hossain

**Affiliations:** 1 Department of Microbiology, University of Dhaka, Dhaka, 1000 Bangladesh; 2 Department of Fisheries, University of Dhaka, Dhaka, 1000 Bangladesh; 3 Present Address: Department of Microbiology, Jagannath University, Dhaka, 1100 Bangladesh

**Keywords:** Cultured shrimp, VP28, Real-time qPCR, Severity, Mutation, Phylogeny

## Abstract

White Spot Syndrome Virus (WSSV), the etiological agent of White Spot Disease (WSD) is a major impediment for shrimp aquaculture in the worldwide. A critical threshold level of WSSV load in infected shrimp is an important trait for disease manifestation and WSSV transmission in cultured shrimp and subsequently make outbreaks. The present study investigated 120 naturally infected cultured shrimp samples by SYBR Green based qPCR assay for WSD diagnosis and quantification of WSSV load. Among them, 94 samples resulted a variable count of WSSV load ranging from 2.1 × 10^8^ to 2.64 × 10^14^ copies/g of shrimp tissue. The severity of WSSV infection was assessed based on the established critical threshold load of WSSV in shrimp tissue. Compared to the established critical threshold value of WSSV load in shrimp tissue, our findings showed the horrifying scenario of the severity of WSSV infection in cultured shrimps of Bangladesh that was found to be above the critical limit to initiate an outbreak in the Bangladeshi shrimp aquaculture industry. The latest phylogenetic pattern was altered from the former monophyletic history among WSSVs of Bangladesh due to a variation at 500th nucleotide of VP28 coding gene. Viruses characterized from recent outbreaks in 2015 and 2017 displayed amino acid substitution at position 167 (G→E) on the surface of VP28 protein which has demonstrated the probable replacement of indigenous virus pool. Therefore, it is imperative to take initiative for the management and prevention of WSSV outbreak to sustain shrimp aquaculture in South-West region of Bangladesh.

**Electronic supplementary material:**

The online version of this article (10.1186/s13568-018-0553-z) contains supplementary material, which is available to authorized users.

## Introduction

White spot syndrome virus (WSSV) is a large dsDNA virus and the causative agent of White spot disease (WSD), has been reported to cause severe mortality in farmed shrimp especially black tiger shrimp, *Penaeus monodon* in many countries (Balasubramanian et al. 2008). Black tiger shrimp is the main coastal aquaculture species and second highest foreign currency earning source in Bangladesh (DoF 2013). Khulna division is the largest farmed shrimp producer (accounting for 86% of national shrimp aquaculture production) and Satkhira district (Southwestern parts of Bangladesh) accounted for 31% of Khulna shrimp farm production and 26% of national shrimp farm production in 2014 (BBS 2017). However, shrimp aquaculture industry in Bangladesh has been greatly suffered from viral diseases particularly WSD and losses hundreds of million dollars every year (Lafferty et al. 2015). Since its first report in East Asia in 1992, WSSV spread quickly to shrimp-farming areas in Southeast Asia, Central and Latin America, causing major economic damage to shrimp aquaculture (Oidtmann and Stentiford 2011). As a result, WSD has been listed a devastating viral disease of crustaceans by the Office of International Epizootic (OIE) (Nunan and Lightner 2011). Cultured penaeid shrimp from post-larvae (PL) to brood stock are highly susceptible to WSSV infection (Vidal et al. 2001).

Cultured shrimp life span is maximum of four to five months. During the culture period, shrimp have to tolerate more variable conditions, less controlled temperature and salinity fluctuation (Jeswin et al. 2015). Extreme salinities is identified as one of the key environmental stress factor that might intensify the susceptibility of shrimp to viral infection (Ramos-Carreño et al. 2014). Therefore, recurrent variation of environmental conditions such as temperature, pH and salinity make shrimp more susceptible to WSSV infection and in such conditions viral load has been found at a very high titer. The viral load in infected shrimp is known to be one of the most important traits for transmission of virus. WSD outbreak is triggered when the viral load in the infected shrimp reached a critical concentration (Durand et al. 2003).

Several studies on WSSV genetic structure and functions have shown that a few envelope proteins are involved in initiation of infection. WSSV genome contains a single copy of VP28 gene (615 bp, 204 amino acids) which encodes for a key structural protein for virion envelope. This envelope protein plays a significant role in progression of systemic infection as it is often involved with viral entry, assembly and budding (Syed Musthaq et al. 2009). It infers that, quantification of copies of this gene in extracted DNA samples from WSSV infected shrimp might be indicative of the number of virions and naked genome prior to assembly present in the infected or carrier host. In Bangladesh, it has been observed from field experience that some farmers in South-West region are able to obtain a reasonably good harvest despite the presence of WSSV infection characterized by severe white spot on carapace, while similar observations have also been reported from China (Lan et al. 2002). These observations suggested the existence of possible disease resistant shrimp and genetic variants of WSSV (Chakrabarty et al. 2014a, b; Mallik et al. 2016; Morse 1994). Genetic variants arisen due to mutations could be a probable reason behind molecular diagnostic failure mainly owing to inhibition of binding PCR primers; that might lead to false negative PCR results or non-specific PCR products which in return, limits the use of PCR based diagnostic kits (Mendoza-cano and Sanchez-paz 2013; Kwok et al. 1990). Thereupon, the comparison of the nucleotide variation in VP28 gene could be considered a possible marker to differentiate WSSV isolates.

Furthermore, our earlier studies were confined to detection, not WSSV load quantification which did not depict the complete scenario of circulating WSSV load in cultured shrimp of Bangladesh (Hossain et al. 2015). Therefore, this study focused for the first time on the quantification of the circulating WSSV load in cultured shrimp samples in this region. Hence, the work reports the first quantitative assay of WSSV infection in naturally infected cultured shrimp to infer the severity of WSD and describes duly the genetic variation of WSSV circulating in the South-West region of Bangladesh.

## Materials and methods

### Shrimp samples collection

The moribund shrimp samples (n = 120) were collected randomly from WSSV prevalent zone of Satkhira district, Bangladesh from January 2014 to June 2017. The sample collection techniques and transportation procedure was done by following the protocol described by Hossain et al. (2015).

### DNA extraction

Total DNA was extracted from tissue samples of WSD suspected shrimps by Maxwell^®^ 16 automated nucleic acid extraction system (Promega, Madison, WI, USA) using Maxwell^®^ 16 tissue DNA purification kit (Promega, Madison, WI, USA; Catalog-AS1030) according to manufacturer’s instruction. Concentration and optical purity of the extracted DNA was evaluated using NanoDrop™ 2000.

### Quantitative real-time PCR (qPCR) assay

Our method was optimized with newly designed primer (WSSV-q28F 5′-TGTGACCAAGACCATCGAAA-3′ and WSSV-q28R 5′-CTTGATTTTGCCCAAGGTGT-3′) following the method developed by Mendoza-Cano and Sánchez-Paz (2013) with some modification (recombinant plasmid based standard instead of purified PCR product based standard). The recombinant plasmid containing VP28 gene (TOPO TA Vector having complete CDS of VP28 gene as an insert) was gel purified using Wizard^®^ SV Gel and PCR Clean-Up System (Promega, Madison, WI, USA). The standard was prepared from serial dilution of purified recombinant plasmid in a linear logarithmic scale of 1.0 × 10^9^ to 10^2^ copies per reaction.

All qPCR reactions were run at a final volume of 25 µL in the Applied Biosystems^®^ 7500 Real-Time PCR system (Foster City, CA, USA) using 2× SYBR^®^ Green PCR Master Mix (Applied Biosystems, Foster City, CA, USA), 100 nM of each of the primers and 2.5 µL (10% of total reaction volume) of each template DNA. Thermal cycling parameters were set for an initial denaturation step at 95 °C for 10 min followed by 40 cycles at 95 °C for 15 s for DNA denaturation with subsequent annealing and extension at 53 °C for 30 s. Melt curve analysis was also performed to differentiate the specific amplicon from primer dimer formation or amplification of other non-specific product. Moreover, the qPCR products were also electrophoresed in agarose gel to nullify the presence of spurious amplicon. The present experiment was conducted with duplicates replication to quantify the viral load.

WSSV load per gram of tissue sample was calculated according to the following equation:

Viral load per gram of tissue = [viral load per reaction × (Final Elution volume/volume of template DNA per reaction) × dilution factor] ± Standard Deviation (SD).

To assess the reproducibility of the standard curve, standard reactions were performed three times independently including duplications of each reaction. The real-time PCR data were analyzed by using 7500 software, version 2.0.6 (Applied Biosystems, Foster City, CA, USA). The data were analyzed by using the statistical program Microsoft Excel 2010 and presented as mean ± SD.

### Polymerase chain reaction amplification and sequencing of VP28

A conventional PCR was performed to amplify the VP28 gene using GoTaq 2× Hot Start Colorless Master Mix (Promega, USA) with forward and reverse primer (Rout et al. 2007). The PCR-amplified products were analyzed by 1% agarose gels containing ethidium bromide. PCR amplicons were purified using the Wizard^®^ SV Gel and PCR Clean-Up System (Promega, USA) and purified PCR products were subjected to an automated cycle sequencing reaction using BigDye^®^ Terminator v3.1 cycle sequencing kit (Applied Biosystems^®^, USA) according to manufacturer’s instructions with the same primers used in the PCR reaction and analysis of the data was performed in ABI Genetic Analyzer (Applied Biosystems^®^, USA).

### Sequence analysis and submission to NCBI GenBank

The raw sequence data were assembled using SeqMan version 7.0 (DNASTAR, Inc., Madison, WI, USA) and the assembled sequences were compared with other entries from NCBI GenBank (Benson et al. 2005) using BLAST (Altschul et al. 1990) search to reveal the identification and matching with VP28 gene of WSSV. Afterwards, assembled sequences of the VP28 gene of WSSV were submitted to NCBI GenBank (Accession No. MF489075–MF489082).

### Assortment of WSSV sequence dataset and rationale behind the dataset generation

A total of 74 sequences were compared in this investigation, among which 18 isolates had origin in Bangladesh (Additional file 1: Table S2). In this study, we have sequenced eight of these isolates from Bangladesh. 56 reference sequences were collected from NCBI GenBank database to represent other countries where WSSV has been identified. We have screened the sequences based on cluster formation in phylogenetic tree to cover each country. After the thorough screening, 42 sequences were selected for final phylogenetic tree reconstruction. In case of mutational analysis, 66 sequences were taken into consideration due to the identical amino acid composition of ten sequences in the dataset. These sequences were generated from the isolates of same location and collection year (Chidambaram/M(3–8)/India/2009, Kadalur-NM(1–4)/India/2009) as presented in Additional file 1: Table S2. The rationale was taken into account to produce a non-redundant dataset by screening which was necessary to leverage the final analytical processes and for better view of the generating results.

### Recombination detection

We first analyzed the sequences of WSSV with RDP v4.56 program (Martin et al. 2015) that incorporates RDP (Martin and Rybicki 2000), GENECONV (Padidam et al. 1999), Bootscan (Martin et al. 2005), Chimaera (Hall 1999), MaxChi (Garcia-Boronat et al. 2008), SiScan (Kabat et al. 1977) and 3Seq (Boni et al. 2007) methods as well as GARD (Kosakovsky Pond et al. 2006) method of Datamonkey webserver (Delport et al. 2010) to find the evidence for recombination events to prevent potential biases during phylogenetic reconstruction. We considered significant events executed by four or more methods using the Bonferroni (Bland and Altman 1995) correction to nullify the false positive results (Faye et al. 2014).

### Phylogenetic tree reconstruction

jModelTest (version 2.1.10) package (Darriba et al. 2012) was used for substitution model selection by computing likelihood score out of 56 models. The best fitted model was selected using lowest corrected Akaike information criterion (cAIC) (Hurvich and Tsai 1993) and Bayesian information criterion (BIC) values (Schwarz 1978). MEGA7 (Kumar et al. 2016) was also implied to check out the best substitution model for the dataset based on AIC (Akaike 1973) and BIC values. Maximum Likelihood (ML) method (Felsenstein 1981) was implied to reconstruct the phylogenetic tree. Fewer than 5% alignment gaps, missing data, and ambiguous bases were allowed at any position as all the sequences were not completely aligned on full range. To observe the statistical significance, 1000 times replication of the branches of the tree was performed.

### Mutational analysis

Variability scores in both nucleotide and amino acid level were calculated using MEGA7 and Seaview v4.6 (Gouy et al. 2009) in both overall dataset including all the sequences and only Bangladesh originated isolates. BioEdit Sequence Alignment Editor v7.2.5 (Hall 1999) was used for a better visualization of the mutations with the position keeping all the sequences altogether in a single frame. Protein Variability server (PVS) (Garcia-Boronat et al. 2008) was used for finding out protein variability index using the Wu-Kabat variability coefficient of the aligned protein sequences (Kabat et al. 1977). Using the PVS, a consensus sequence was also obtained from this alignment that also search for the variable site with a threshold level of less than or equal to 1. Then, Skylign (Wheeler et al. 2014) was used to generate sequence logo after inputting the aligned file. In case of Skylign, fragmented sequences were taken into account and the probability of amino acid residue for each position was detected.

### Protein modeling and alignment

On the basis of major mutation in VP28 isolated from Bangladesh, the protein modeling was performed taken PDB id 2ED6 (Tang et al. 2007) and BAN_SH_BG-1C_2014 as template and target, respectively. SWISS-MODEL server (Biasini et al. 2014) was used for generating the protein model and then PyMol (DeLano 2002) was used for visualization of the PDB file. Using PyMol, alignment of the Bangladeshi isolate and the reference VP28 (2ED6) focusing on the region (160–170). Using PDB files of target, Ramachandran plot (Ramachandran et al. 1963) was produced to verify whether the residues were in one of the three regions-favored, allowed and outlier using RAMPAGE (Chen et al. 2010). To assess both the quality and energy criteria of 3D structure, PROSA (Wiederstein and Sippl 2007; Sippl 1993) was used. The 3D structures of the reference template sequence (PDB id—2ED6) and Bangladeshi isolate sequence (BAN_SH_BG-1C_2014) were superimposed on each other to visualize the mutational change in a clear view by implying PyMol.

## Results

### Quantification of WSSV in cultured shrimps

**Fig. 1:**
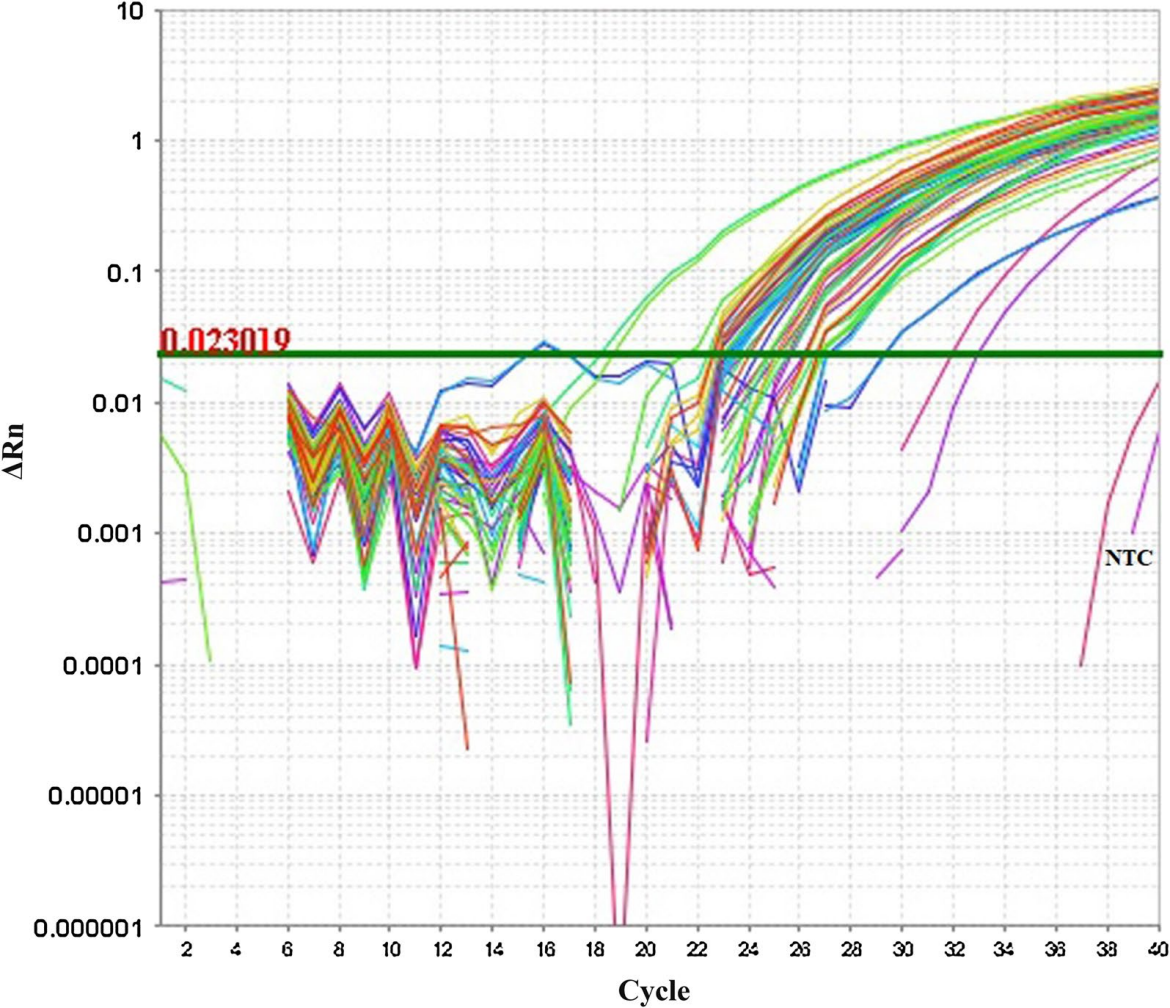
Amplification plots of WSSV positive samples and no template control (NTC). The amplification of positive samples was confirmed by significant fluorescent signal overhead the base line. The significant fluorescent signal above the baseline was not spotted for the negative control (NTC). The C_T_ (cycle threshold) value of NTC was beyond the determination index

**Fig. 2:**
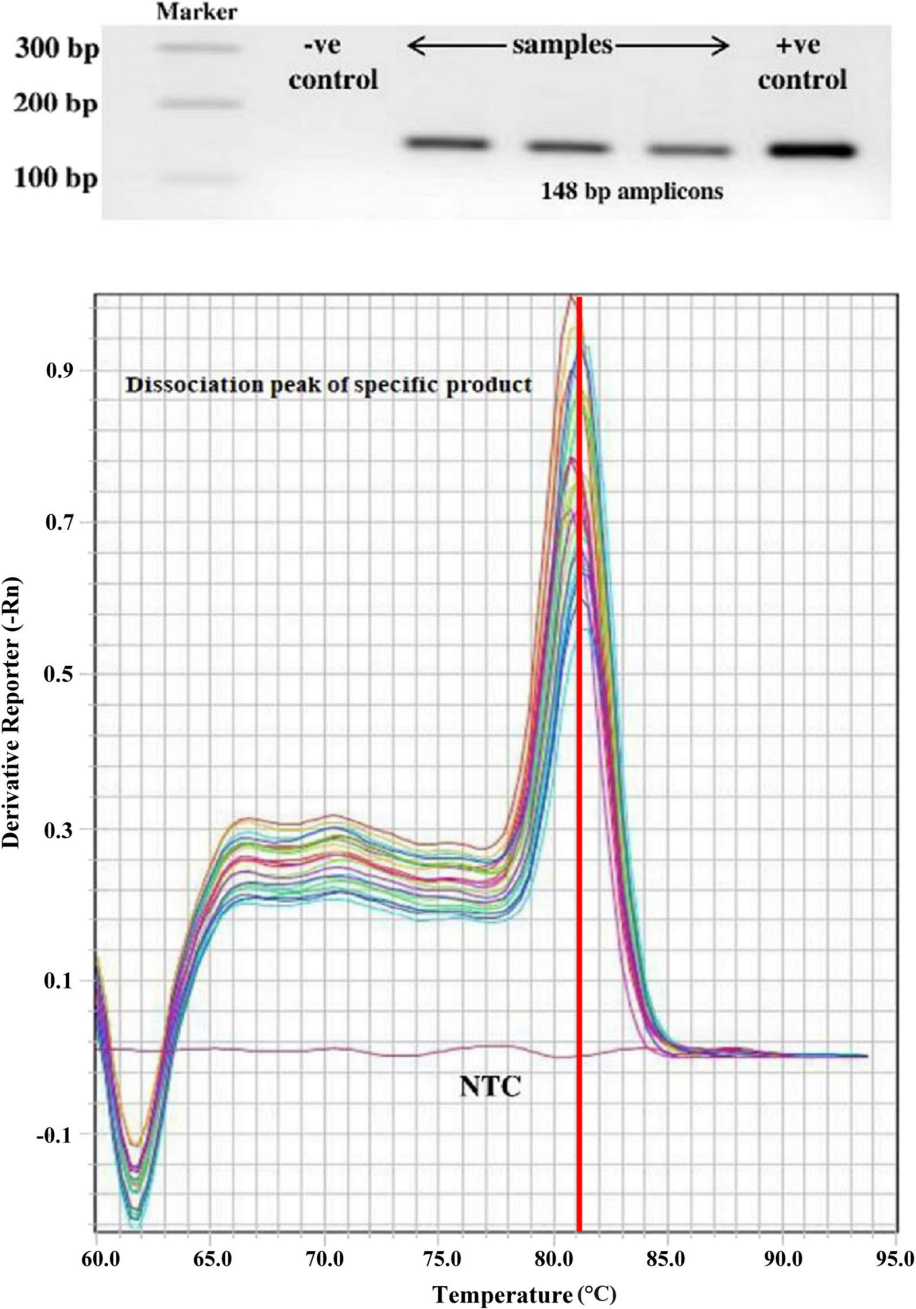
Specific dissociation peak of WSSV positive samples by melt curve analysis with corresponding conventional PCR amplicon (148 bp) targeting VP28 gene of WSSV. The ethidium bromide-stained agarose gel (1.5%) was used to visualized 148 bp PCR amplicon generated by qVP28F and qVP28R primers. The Tm value (81.1 ± 17 °C) of real-time PCR specified the unified WSSV amplicon in the samples

To quantify viral loads, 120 shrimp samples from 120 different farms were tested (from January 2014 to June 2017). Positive amplification pattern was observed in 94 samples with the presence of specific PCR product of 148 bp confirmed by significant fluorescence signal having a signature asymptotic curve (Fig. 1). No significant fluorescence signal was observed for the duplicated negative control (NTC) and 26 negative samples (compared to standard positive samples). The cycle threshold value (C_T_) for the negative samples and negative control of amplification was beyond determination index (three times of independent real-time PCR assay with duplication in each sample). On the other hand, cycle threshold value (C_T_) in all 94 positive samples were between 18 and 33, and all positive samples showed quantitative index in real-time qPCR assay. After amplification, all samples were subjected to melt curve analysis. Melting temperatures of amplicons (Tm) were presented by plotting the values of negative derivation of the fluorescence signal (-Rn) against temperature in degree Celsius. Melt curve analysis of standard (recombinant plasmid based) and WSSV positive field samples (crude DNA) yielded single expected dissociation peak of Tm at 81.11 ± 0.17 °C, indicating specific amplification of target gene (Fig. 2). Another Tm peak (ranged from 65 to 67° C) was evident beyond the expected one after melt curve analysis especially of the negative samples which suggests the presence of possible primer dimer. The agarose gel electrophoresis of the qPCR products nullified the presence of spurious amplicon other than the primer dimer (in case of negative samples).

### WSSV load and severity of infection in cultured shrimps of Bangladesh

Quantitative study revealed a wide range of WSSV load (8.7 × 10^3^ to 1.10 × 10^10^ copies; mean value) per 2.5 µL of template DNA which is equivalent to 2.1 × 10^8^ to 2.64 × 10^14^ copies/g of tissue (Additional file 1: Table S1). Among 94 positive samples, 87 samples were found to contain WSSV load between the logarithmic scales of 9 and 11. However, WSSV load in two samples were on the logarithmic scale of 12. In addition, one sample contained WSSV load at 14 logarithmic scale (phenotypic observation: highly spotted on entire exoskeleton) and WSSV load of 8 logarithmic scale was observed for another four samples (phenotypic observation: spot not appeared on exoskeleton of shrimp).

The degree of WSSV infection was categorized based on the established critical threshold limit (Table 1) described by Walker et al. (2011) and Tang and Lightner (2000). According to Walker et al. (2011), out of 120 shrimp samples, 94 were WSSV positive in which 73 (77.66%) were graded as moderate to heavy infection and 21 (22.34%) were grouped as light or very light infections (Table 1). In another context based on the report of Tang and Lightner (2000), the number of WSSV genomes increased over 100-fold between severity levels (Table 1). The severity of infection level G3 (moderate to severe) and G4 (severe) was found in 87 (72.5%) samples; altogether 78.33% samples were infected which stipulates the alarming situation of WSD in the South-West region of Bangladesh.

**Table 1:** The potential severity of WSSV infection in farmed shrimp was evaluated by the laboratory based severity study of Walker et al. (2011) and Tang and Lightner (2000)

Category of Severity of WSSV infection in Shrimp		Severity of WSSV infection in shrimps from January 2014 to June 2017	
C_T_ value	Level of infection	Total (n = 120)	% of samples
According to Walker et al. (2011)			
< 24.33	Heavy	37	30.83
28.33–24.33	Moderate	36	30
31.52–28.33	Light	11	9.17
34.82–31.52	Very little	10	8.33
> 34.82	Negative	26	21.67

WSSV load/g shrimp tissue	Severity level	Total (n = 120)	% of samples
According to Tang and Lightner (2000)			
2.0 × 10^5^	G1-mild	0	0
2.0 × 10^7^	G2-mild to moderate	7	5.83
2.0 × 10^9^	G3-moderate to severe	74	61.67
2.0 × 10^11^	G4-severe	13	10.83
No load	Negative	26	21.67

### Compiled dataset for phylogenetic and mutational analyses

Altogether 74 sequences were taken into account in this study where 18 of these were isolated from Bangladesh considering the location and year of isolation (Additional file 1: Table S2). Among the isolates, 66 of the sequences were chosen for mutational analysis.

### Observation of phylogenetic history

**Fig. 3:**
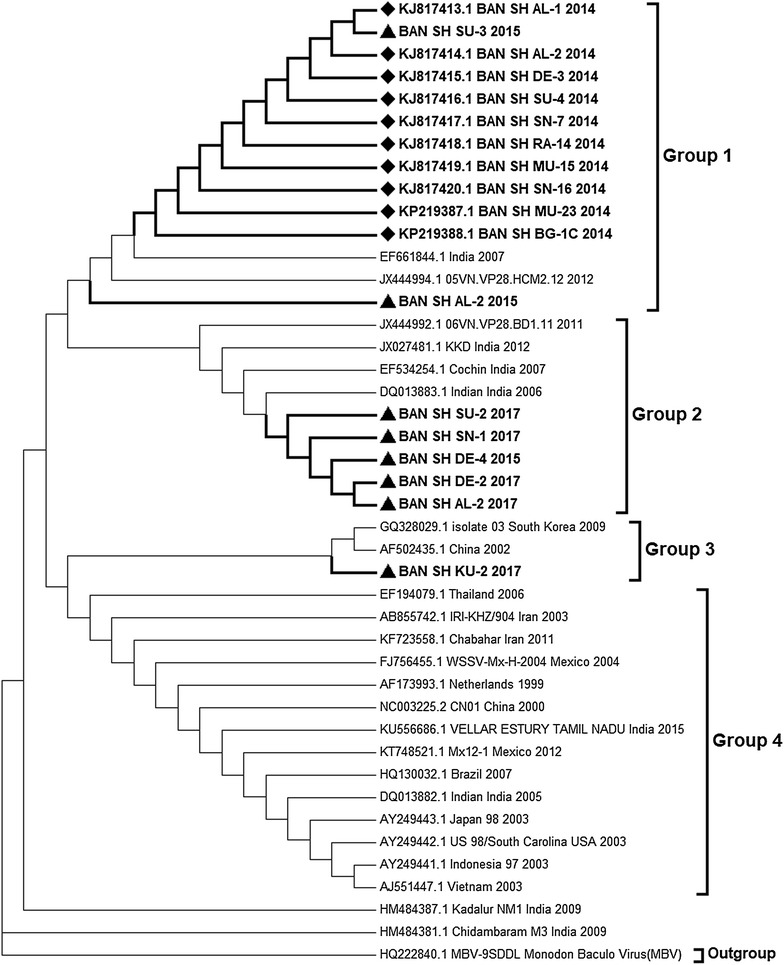
Molecular phylogenetic analysis (codon based) of WSSV *isolated* from Bangladesh between 2014 to 2017. Nucleotide sequences of VP28 coding region were used to construct the tree based on the Tamura-Nei model by Maximum Likelihood method. The analysis included 42 nucleotide sequences among which 18 were from Bangladesh. The sequences generated from isolates of Bangladesh are shown in bold letter, which are placed in three distinct clusters among four. Sequences generated exclusively for this study and submitted from previous study (Hossain et al. 2015) are marked with (filled triangle) and (filled diamond) symbols, respectively. Monodon Baculovirus (Accession No. HQ222840) was taken as outgroup in the tree

RDP and GARD based recombination detection methods implemented in this study showed no breakpoint event and hence recombination in VP28 sequences along with out-group [Monodon Baculovirus (MBV)] included for further phylogenetic studies. Tamura-Nei substitution model with uniform rate variation and pattern was chosen as best model based on AIC, cAIC and BIC values generated from jModelTest and MEGA7 softwares. The isolates from Bangladesh could be divided into different clusters as shown from the tree (Fig. 3). Two clusters (group 1 and 2) contained 17 of the sequences isolated from Bangladesh that mostly related to isolates from India and Vietnam. Another cluster contained only one isolate from Bangladesh (BAN_SH_KU-2_2017) along with isolates from other countries. The other countries included South Korea, China, Thailand, Iran, Mexico, Netherlands, India, Brazil, Japan, USA, Indonesia and Vietnam.

The isolate BAN_SH_SU-3_2015 clustered with other VP28 sequences that was previously reported (Hossain et al. 2015) to be circulated in Bangladesh. BAN_SH_KU-2_2017 with the isolates of China and South Korea formed a slightly different branch within group 3. VP28 gene sequences of other local samples (BAN_SH_SU-2_2017; BAN_SH_SN-1_2017; BAN_SH_DE-2_2017; BAN_SH_AL-2_2017; BAN_SH_DE-4_2015; BAN_SH_AL-2_2015) clustered together in group 2.

### Analysis of mutation pattern in VP28

In total, there were found 13 mutations (Additional file 2: Figure S1) covering all the isolates from different countries specially focusing on and clustering the Bangladeshi isolates serially for better visualization of the dissimilarity with other sequences of VP28 (Table 2). White spot syndrome virus strain CN01 (Accession No. NC_003225) was used as reference sequence for mutational analysis unambiguity. Amino acid residues at positions 42 and 167 showed mutations in case of Bangladeshi isolates among which position 167 showed glycine instead of glutamic acid for the isolates circulating in 2014 (Hossain et al. 2015) and only one isolate from 2015 (BAN_SH_SU-3_2015) (Additional file 3: Figure S2).

**Table 2:** Amino acids in the mutated positions showing the percentage along with position and region name of WSSV VP28

Position	Amino acids (%)	Region in VP28	Isolate of mutated sequence
38	E (98.4), V (1.6)	α1	Thailand 2006
40	H (95.4), R (3.1), P (1.5)	α1	China 2002, China-95/Dalian China 2003 (R), Isolate-03/South Korea/2009 (P)
*42*	*D (63.1), G (36.9)*	*α1*	*Bangladesh (2014*–*17), India (2006*–*07, 09, 12), Vietnam (2011*–*12)*
76	T (98.5), A (1.5)	β2–β3	05VN.VP28.HCM2.12_Vietnam_2012
114	Q (97), R (3)	β4–β5	Kadalur-NM1_India_2009 Chidambaram-M3_India_2009
124	V (98.5), M (1.5)	β5	India 2007
135	T (98.5), A (1.5)	β6	07VN.VP28.BD2.12_Vietnam_2012
145	P (98.5), L (1.5)	β6–β7	Thailand 2006
162	S (98.5), F (1.5)	β7	India 2003
*167*	*E (83.3), G (16.7)*	*β7*–*β8*	*Bangladesh 2014 (All Isolates), BAN/SH/SU*-*3/2015*
173	C (98.5), R (1.5)	β8	05VN.VP28.HCM2.12_Vietnam_2012
179	A (98.5), V (1.5)	β8	China 2006
182	A (98.5), E (1.5)	β9	Chabahar_Iran_2011

The country name and year of isolation of the isolates that showed the mutation from the Reference Sequence CN01/China/2000 (Accession No.—NC_003225)

Italicized data indicate the specific amino acid variations (frequency) with respective positions and regions in VP28 of WSSV isolates collected from Bangladesh and related countries

### Prediction and mapping of exclusive mutation found in Bangladesh

**Fig. 4:**
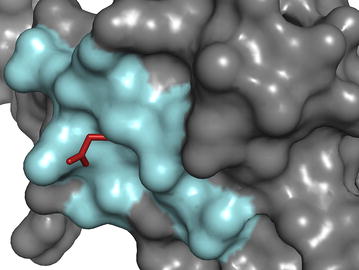
Three-dimensional structure of aligned WSSV VP28 envelope protein of BAN_SH_BG-1C_2014 that was taken as representative of isolates collection in 2014 in Bangladesh and PDB id 2ED6. The side chain of Glutamic acid (E) in the reference protein protruded out of the surface portion where the BD isolate contained Glycine (G). The amino acid positioned between the beta-strands seven and eight. The red color in the figure showed the protruded side chain. Paste color and gray color represented the target region (160–170 amino acid) and the rest of the protein. Surface and line style was used to visualize the protein and side chain of glutamic acid

After producing PDB file, the quality of the structure was checked using Ramachandran plot (Additional file 4: Figure S3) showing that 96.4% of the residues fell within the favored region. The PROSA analysis of the model showed maximum residues to have negative interaction energy, and the overall Z-score is − 6.4. In the superimposed structure, the region flanking (10 bp) the unique mutation (E→G) was focused (Fig. 4) and the mutated amino acid side chain protruded out of the structure. This position is in between β-barrel no. seven and eight (β7–β8).

## Discussion

### Higher severity indicates WSD outbreak in South-West region of Bangladesh

Satkhira is the top-tier shrimp producing district (BBS 2017), therefore, it is imperative to conduct regular surveillance of WSSV in this area. Our optimized SYBR Green qPCR method was applied to diagnose and quantify WSSV load in diseased cultured shrimp samples. In our previous studies, conventional and real-time PCR based detection of WSSV in cultured shrimp was reported from Bangladesh (Hossain et al. 2015, 2016). In this study, quantitative real-time PCR assay was performed on field shrimp samples; to reduce the possible PCR inhibitors, all samples were diluted one logarithmic scale which is supported by a former study (Leal et al. 2013). The resulted standard deviation value of Tm (± 0.17) °C revealed significant agreement in the amplified PCR products (Mendoza-Cano and Sánchez-Paz 2013).

Infections at low or even moderate to high intensities do not necessarily result in disease, however, once the virus reaches a critical threshold level in some shrimp, disease might occur (Walker et al. 2011). Once some shrimp in a pond get infected with WSSV, other shrimp in that pond will be exposed to high level of virus through ingestion or immersion and quickly develop severe disease resulting in rapid production failure. Presence of minimum 10^5^ WSSV copies is necessary to transmit WSSV in shrimp by immersion in cultured condition (Meng et al. 2010). A recent report demonstrated that 10^7.1^–10^7.5^ virus per gram of tissue is needed to infect shrimp by peroral inoculation and via feeding (Thuong et al. 2016). A study by Jeswin et al. (2015) has demonstrated that a dose of 6 × 10^7^ WSSVg^−1^ was sufficient to kill shrimp effectively within 96 h. Analysis of WSSV titer in diseased shrimp under current study, showed that all 94 WSSV positive shrimp samples had viral load above 10^8^ copies/g of tissue. This high viral loads might pave a way to transmit viruses to non-infected cultured shrimp. With poor pond management, the virus causing the disease outbreak can also spread to adjacent ponds in a radiating outbreak, primarily through the transfer of WSSV contaminated water (Walker et al. 2011). In addition, our personal experiences, farmer’s information and observed field scenario also confirmed WSD outbreak in the present study areas.

Sahoo et al. (2010) have reported that moderate or heavy level of WSSV presence in ponds is significantly associated with production outcomes in terms of survival, yield and length of the production cycle. Furthermore, the greater the level of severity of WSSV infection, the faster the rate of mortality. In the study area, most farms practice improved extensive or semi-intensive culture technology and do not follow scientific management. Tidal water is filled in, and drained out, by using the same structure as inlet and outlet of the farm. The water enters into the pond was found to mix with the discharge of the same farm or other farms that would contaminated the water. These factors, collectively, might be attributed as one of the most key sources of WSSV infections in cultured shrimps in Bangladesh.

Till date, we have no sustainable treatment technique available for extermination of WSSV. WSSV infected shrimp cannot be cured, only preventive measures could be taken to get rid of this burden. The whole culture environments might be improvised by implementing good aquaculture practices (GAqP) and regular surveillance of critical WSSV load (a load that implies disease outbreak) using quantitative real-time PCR technique that might resist mass mortality due to WSSV infection and huge economic destruction from unexpected outbreak of this disease in Bangladesh. In our previous report (Hossain et al. 2015), we presented the high prevalence of WSSV in shrimp farming areas of Bangladesh from where the field samples were collected. Quantitative index of high WSSV titer observed in the current study might be a key underlying cause of the WSD outbreaks in South-West region of Bangladesh.

### One single nucleotide change has altered the phylogenetic pattern among Bangladeshi isolates

In DNA virus, rates of evolutionary change in genome is slow and the nucleotide substitution rate is far lower than RNA virus (Duffy et al. 2008). Being a DNA virus, WSSV must show the properties of being conserved in both nucleotide and protein level. In case of VP28 of WSSV, the major structural protein coding gene displays conservation of the DNA sequence, even at a level of 99–100% in most cases (You et al. 2004; Molina‐Garza et al. 2008).

In Bangladesh, the isolated viruses showed genetic divergence falling under different clusters. These different clusters consisted of WSSV samples from other countries. From the phylogenetic tree, it can be assumed that all of the isolates collected in 2014 from Bangladesh were quite diverged from the samples collected in the following years and the result correlates with the previous work (Hossain et al. 2015). Along with the 2014 isolates, two isolates from 2015 clustered in the same group- BAN_SH_SU-3_2015 and BAN_SH_AL-2_2015. On the other hand, isolates from 2017 got divided into two groups wherein BAN_SH_KU-2_2017 fell under separate clade from the other four isolates. The sequences from India, Vietnam and Bangladesh fell into all of the major clades, that is not found in case of other countries as shown in the tree.

A more conservative approach to phylogeny might give a different result from the one shown in this study. However, the divergence was not that significant in the reconstructed phylogenetic tree as there have no significant variations present among the sequences of VP28. In case of Bangladeshi isolates, only one nucleotide got changed that means 0.3% of the total nucleotide changed. Interestingly, this single change has a major impact on the protein variability of VP28. We have found by calculating the diversity both in nucleotide and protein level among sequences from different geographical locations that 5% and 7.7% of the nucleotides and amino acids were variable.

The conservation of sequences of the coding region of structural proteins of different WSSV isolates provides a strong basis for generating highly specific and sensitive nucleic acid and immuno-based molecular detection methodologies for WSSV (You et al. 2004). As an example, we have used PCR primers designed based on the sequence of VP28 gene for detection of WSSV. Based on the sequence homology or similarity, complete VP28 protein can be applied as potential recombinant antigen to produce vaccine that triggers the adaptive quasi-immune response in shrimp (Namikoshi et al. 2004, Venegas et al. 2000). However, all WSSV proteins involved in viral infection of shrimp need to be assessed in molecular level to uncover the detailed genetic make-up.

### The first time report of mutation pattern of VP28: an exclusive mutation was spotted

The shrimp (*Penaeus monodon)* protein PmRab7 is located in endosome that acts as receptor for the WSSV envelop protein VP28 (Sritunyalucksana et al. 2006; van Hulten et al. 2001). VP28 is a major structural envelope protein of WSSV responsible for systemic infection in shrimp forming an important part of “infectome” that is found to be crucial in cell recognition, attaching and penetration into the shrimp cell (Chang et al. 2010; van Hulten et al. 2001). VP28 is located on the outer surface of the virus and the transmembrane N-terminal portion of the protein embedded in the envelope followed by the N–terminal α–helix (α1) and then C–terminal nine stranded β–barrel structure-a feature uncommon in other viral proteins (Tang et al. 2007).

From the result of mutational analysis (summarized in Table 2), some unique mutations at different geographical locations were found. Joseph et al. (2015) reported the nucleotide and amino acid variations found in VP28 of WSSV. For example, two unique mutations were captured from the isolates of Thailand in 2006. The positions of the mutated amino acids are in 38 and 145, respectively among which P→L substitution might be crucial as the amino acid situated in the outside of the envelope in a beta-strand (Tang et al. 2007). In case of Bangladesh, the unique mutation (E→G) at position 167 that falls between two beta strands of protein that are thought to be involved in receptor recognition (Verma et al. 2013). This exclusive mutation was found in the isolates of Bangladesh collected in 2014 and only in one of the isolates of 2015. Another mutation at position 42 (D→G) was found common to all of the isolates of WSSV in Bangladesh. The significance of these mutations in the particular positions of VP28 was not determined as none of the amino acid was found to interact with PmRab7 of shrimp (Verma et al. 2013).

For finding out the exact position and possible role of the amino acids at the uniquely mutated position in a 3D structure of VP28, we performed the alignment of the designed and representative VP28 protein from Bangladesh over the reference protein from PDB consisting of different amino acid other than that was found for the first time in Bangladesh. The region spanning (160–170) amino acid residues positioned on the surface area rather than groove area of VP28 protein (Fig. 4). The change of amino acid (glutamic acid to glycine, E→G) at position 167 may not give any positive effect on the infectivity of VP28 as it does not give much opportunity to interact with other proteins or ions rather than glutamic acid. Further investigation into the interaction of the mutated amino acid with different related proteins or ions will shed light on the possible role.

To reduce the risks of WSD outbreak in shrimp farming, we recommend to stock virus-free PL produced from PCR-negative broods and proper maintenance of post-stocking to reduce risks of WSSV infection. Therefore, quantitative Real-time PCR methods can be applied to detect the infected brood shrimp or PL prior to infestation of the virus to its school that results an outbreak. From our observation, it can be suggested that lack of proper quarantine system on import of fish/shrimp and fish/shrimp brood from the WSSV endemic country could be responsible for a WSD outbreak in Bangladesh.

The higher WSSV load in our studied samples which is found above the critical limit for dissemination of the virus can subsequently lead to initiate an outbreak. This study describes a unique mutation pattern of VP28 gene of circulating WSSV in South-West region of Bangladesh. Overall, the findings of this research work could be a beacon for rapid screening of WSSV (including the new mutant) in brood and post-larvae of shrimp and may be a way out to mitigate the huge economic loss every year in shrimp farming of Bangladesh.

## Additional files

**Additional file 1: Table S1.** qPCR profile of the studied samples by our designed and standardized method. **Table S2.** Accession no., sequence IDs, sources and collection time of the WSSVs sequences of dataset.

**Additional file 2: Figure S1.** Wu-Kabat protein variability index showed 13 unique mutations as observed from the protruded peaks’ positioned along with the amino acid residue of VP28 protein.

**Additional file 3: Figure S2.** The aligned protein sequence data of VP28 taking into consideration 66 sequences generated by BioEdit Sequence Alignment Editor. The left portion of the frame contained the isolate ids of the sequences. The digit in the upper portion of the figure represented the residue number of protein. The red bar indicates the unique mutation position along with the mutated amino acid.

**Additional file 4: Figure S3.** Ramachandran plot of modeled VP28 protein of WSSV. The number of residues in favored, allowed and outlier region are 96.4, 3.6 and 0%, respectively. On the X and Y axes, ϕ (phi) and ψ (psi) represent the torsion angles around Cα (alpha carbon) to amine and carboxyl group of different amino acids.

## Abbreviations

WSSV: white spot syndrome virus
WSD: white spot disease
OIE: office of international epizootic
NCBI: national center for biotechnology information
BLAST: basic local alignment search tool
cAIC: corrected akaike information criterion
BIC: Bayesian information criterion
AIC: Akaike information criterion
ML: maximum likelihood
PDB: protein data bank
3D: three dimensional
C_T_: cycle threshold
Tm: melting temperature
MBV: *Penaeus monodon*-type baculovirus
P: proline
L: lysine
E: glutamic acid
G: glycine
D: aspartic acid
