# Supplementary material for: Circulatory white spot syndrome virus in South-West region of Bangladesh from 2014 to 2017: molecular characterization and genetic variation
Source: AMB Express. 2018 Feb 20;8:25. doi: 10.1186/s13568-018-0553-z (PMC5818386; doi:10.1186/s13568-018-0553-z)
Supplement: Supplementary file 1 — Additional file 1: Table S1. qPCR profile of the studied samples by our designed and standardized method. Table S2. Accession no., sequence IDs, sources and collection time of the WSSVs sequences of dataset. [file 13568_2018_553_MOESM1_ESM.docx]

**Table S1:** qPCR profile of the studied samples by our designed and standardized method.

| **Sample Id** | **qPCR Profile** | | | | |
| --- | --- | --- | --- | --- | --- |
|  | **C_T_** | **WSSV load per reaction**  **(**$\bar{\boldsymbol{X}}$**)** | **Mean of WSSV load per gram of tissue sample (**$\bar{\boldsymbol{X}}$**)** | **Standard deviation (SD)** | **Tm of the amplified Product (ºC)** |
| BAN_SH_MU-1_2014 | 25.823 | 2.53E+05 | 6.07E+09 | 8.71E+08 | 80.841 |
| BAN_SH_MU-2_2014 | 26.149 | 2.17E+05 | 5.20E+09 | 1.40E+09 | 81.210 |
| BAN_SH_MU-3_2014 | 26.695 | 1.62E+05 | 3.90E+09 | 8.11E+08 | 81.303 |
| BAN_SH_MU-4_2014 | 26.638 | 1.57E+05 | 3.77E+09 | 2.80E+08 | 80.748 |
| BAN_SH_MU-5_2014 | 23.818 | 7.07E+05 | 1.70E+10 | 1.11E+09 | 80.841 |
| BAN_SH_MU-6_2014 | 25.987 | 2.32E+05 | 5.56E+09 | 4.63E+08 | 81.210 |
| BAN_SH_MU-8_2014 | 29.117 | 4.64E+04 | 1.11E+09 | 1.35E+08 | 81.210 |
| BAN_SH_MU-9_2014 | 25.972 | 2.43E+05 | 5.83E+09 | 2.31E+09 | 81.026 |
| BAN_SH_MU-10_2014 | 26.338 | 1.94E+05 | 4.65E+09 | 4.73E+08 | 81.026 |
| BAN_SH_MU-13_2014 | 25.158 | 3.54E+05 | 8.51E+09 | 4.24E+08 | 81.210 |
| BAN_SH_MU-15_2014 | 27.430 | 1.14E+05 | 2.72E+09 | 9.38E+08 | 81.026 |
| BAN_SH_BU-17_2014 | 26.766 | 1.69E+05 | 4.05E+09 | 6.67E+08 | 81.297 |
| BAN_SH_BU-18_2014 | 23.157 | 1.14E+06 | 2.73E+10 | 3.02E+09 | 81.297 |
| BAN_SH_BU-20_2014 | 22.549 | 1.57E+06 | 3.77E+10 | 4.39E+09 | 80.941 |
| BAN_SH_BU-22_2014 | 22.698 | 1.45E+06 | 3.48E+10 | 3.34E+09 | 81.297 |
| BAN_SH_BU-24_2014 | 22.890 | 1.33E+06 | 3.20E+10 | 8.95E+09 | 80.941 |
| BAN_SH_BU-25_2014 | 22.563 | 1.59E+06 | 3.81E+10 | 1.09E+10 | 80.940 |
| BAN_SH_BU-26_2014 | 23.572 | 9.22E+05 | 2.21E+10 | 5.01E+09 | 81.208 |
| BAN_SH_BU-27_2014 | 25.292 | 3.66E+05 | 8.79E+09 | 6.51E+08 | 81.297 |
| BAN_SH_BU-30_2014 | 32.416 | 8.75E+03 | 2.10E+08 | 8.26E+07 | 81.119 |
| BAN_SH_BU-31_2014 | 26.071 | 2.42E+05 | 5.82E+09 | 3.16E+08 | 81.030 |
| BAN_SH_BU-32_2014 | 27.01 | 1.47E+05 | 3.54E+09 | 1.51E+08 | 81.297 |
| BAN_SH_BU-33_2014 | 23.526 | 9.33E+05 | 2.24E+10 | 9.33E+08 | 81.208 |
| BAN_SH_BU-34_2014 | 21.717 | 1.96E+06 | 4.71E+10 | 3.84E+09 | 81.119 |
| BAN_SH_RN-35_2014 | 27.476 | 1.15E+05 | 2.76E+09 | 1.36E+08 | 81.297 |
| BAN_SH_RN-36_2014 | 24.432 | 6.05E+05 | 1.45E+10 | 6.19E+09 | 81.119 |
| BAN_SH_RN-38_2014 | 24.043 | 7.23E+05 | 1.74E+10 | 4.81E+09 | 81.297 |
| BAN_SH_RN-43_2014 | 23.356 | 1.02E+05 | 2.45E+10 | 1.06E+09 | 81.297 |
| BAN_SH_RN-44_2014 | 25.336 | 3.58E+05 | 8.58E+09 | 4.84E+08 | 80.980 |
| BAN_SH_RN-45_2014 | 22.525 | 1.60E+06 | 3.85E+10 | 7.97E+09 | 80.94 |
| BAN_SH_RN-47_2014 | 24.492 | 5.76E+05 | 1.38E+10 | 4.75E+09 | 80.941 |
| BAN_SH_RN-48_2014 | 24.801 | 4.75E+05 | 1.14E+10 | 3.98E+08 | 81.297 |
| BAN_SH_RN-49_2014 | 23.422 | 9.87E+05 | 2.37E+10 | 2.01E+09 | 81.297 |
| BAN_SH_RN-50_2014 | 18.28 | 7.35E+07 | 1.76E+12 | 4.24E+10 | 81.119 |
| BAN_SH_MU-11_2014 | UND | - | - | - | 65.580 |
| BAN_SH_MU-14_2014 | UND | - | - | - | 66.741 |
| BAN_SH_BU-28_2014 | UND | - | - | - | 66.026 |
| BAN_SH_BU-29_2014 | UND | - | - | - | 66.116 |
| BAN_SH_RN-40_2014 | UND | - | - | - | 67.187 |
| BAN_SH_.RN-42_2014 | UND | - | - | - | 66.116 |
| BAN_SH_AS _01_2015 | 21.319 | 1.07 E+07 | 2.57 E+11 | 4.62E+10 | 80.866 |
| BAN_SH_AL-2_2015 | 20.846 | 1.37 E+07 | 3.29 E+11 | 7.18E+10 | 81.446 |
| BAN_SH_AL _05_2015 | 21.746 | 8.50 E+06 | 2.04 E+11 | 3.46E+10 | 81.446 |
| BAN_SH_AL _07_2015 | 20.951 | 1.31 E+07 | 3.14 E+11 | 5.89E+10 | 81.446 |
| BAN_SH_AS _08_2015 | 21.797 | 8.27 E+06 | 1.98 E+11 | 2.62E+10 | 81.059 |
| BAN_SH_AS _09_2015 | 21.051 | 1.24 E+07 | 2.97 E+11 | 2.56E+10 | 80.866 |
| BAN_SH_AS _10_2015 | 20.41 | 1.75 E+07 | 4.21 E+11 | 4.62E+10 | 80.673 |
| BAN_SH_AS _11_2015 | 21.087 | 1.22 E+07 | 2.92 E+11 | 3.31E+10 | 80.866 |
| BAN_SH_AS _12_2015 | 18.37 | 7.28 E+07 | 1.75 E+12 | 2.89E+11 | 81.119 |
| BAN_SH_DE _3_2015 | 23.191 | 3.89 E+06 | 9.33 E+10 | 7.39E+09 | 81.446 |
| BAN_SH_DE-4_2015 | 22.216 | 6.59 E+06 | 1.58 E+11 | 1.46E+10 | 81.446 |
| BAN_SH_DE _16_2015 | 22.436 | 5.85 E+06 | 1.40 E+11 | 2.31E+10 | 81.446 |
| BAN_SH_DE _18_2015 | 22.82 | 4.75 E+06 | 1.14 E+11 | 1.16E+10 | 81.253 |
| BAN_SH_DE _19_2015 | 22.646 | 5.22 E+06 | 1.25 E+11 | 2.13E+10 | 81.253 |
| BAN_SH_DE _20_2015 | 22.835 | 4.71 E+06 | 1.13 E+11 | 1.01E+10 | 81.446 |
| BAN_SH_DE _21_2015 | 25.395 | 1.18 E+06 | 2.82 E+10 | 7.81E+09 | 81.253 |
| BAN_SH_DE_ 22_2015 | 21.765 | 8.41 E+06 | 2.02 E+11 | 6.23E+10 | 81.059 |
| BAN_SH_DE _23_2015 | 25.047 | 1.42 E+06 | 3.41 E+10 | 3.38E+09 | 80.866 |
| BAN_SH_DE _24_2015 | 25.469 | 3.51 E+05 | 8.43 E+09 | 7.16E+08 | 80.941 |
| BAN_SH_SN_ 25_2015 | 23.059 | 1.21 E+06 | 2.93 E+10 | 1.56E+09 | 81.059 |
| BAN_SH_SS_ 27_2015 | 31.275 | 4.87E+04 | 1.17E+09 | 1.02E+08 | 81.446 |
| BAN_SH_SU-3_2015 | 32.323 | 1.73 E+04 | 4.14 E+08 | 4.07E+07 | 81.250 |
| BAN_SH_SU _31_2015 | 24.838 | 1.59 E+06 | 3.82 E+10 | 5.16E+09 | 81.446 |
| BAN_SH_SU_ 33_2015 | 32.985 | 1.93 E+04 | 4.6 E+08 | 2.75E+07 | 81.059 |
| BAN_SH_SU_34_2015 | 25.07 | 1.40 E+06 | 3.37 E+10 | 4.19E+09 | 81.446 |
| BAN_SH_SU_35_2015 | 30.522 | 7.32 E+04 | 1.76 E+09 | 1.56E+08 | 80.480 |
| BAN_SH_TA _39_2015 | 26.254 | 7.39 E+05 | 1.77 E+10 | 1.16E+09 | 81.446 |
| BAN_SH_TA _42_2015 | 24.024 | 2.47 E+06 | 5.94 E+10 | 6.28E+09 | 81.059 |
| BAN_SH_TA _43_2015 | 27.271 | 4.85 E+05 | 1.17E+10 | 2.27E+09 | 81.253 |
| BAN_SH_TA _44_2015 | 23.452 | 3.54 E+05 | 8.5E+09 | 7.76E+08 | 81.446 |
| BAN_SH_TA _46_2015 | 27.584 | 3.59 E+05 | 8.63 E+09 | 9.28E+08 | 80.886 |
| BAN_SH_TA _49_2015 | 26.616 | 6.08 E+05 | 1.46 E+10 | 2.23E+09 | 81.059 |
| BAN_SH_TA _50_2015 | 24.818 | 1.59 E+05 | 3.83 E+09 | 3.17E+08 | 81.446 |
| BAN_SH_AL_ 06_2015 | UND | - | - | - | 66.411 |
| BAN_SH_DE _05_2015 | UND | - | - | - | 67.346 |
| BAN_SH_SU_ 32_2015 | UND | - | - | - | 67.128 |
| BAN_SH_SU_36_2015 | UND | - | - | - | 67.137 |
| BAN_SH_TA _40_2015 | UND | - | - | - | 67.539 |
| BAN_SH_TA _41_2015 | UND | - | - | - | 62.325 |
| BAN_SH_TA _48_2015 | UND | - | - | - | 67.513 |
| BAN_SH_MU-03_2016 | 26.631 | 1.67E+05 | 4.00E+09 | 4.22E+08 | 81.210 |
| BAN_SH_BU-09_2016 | 26.637 | 1.8E+05 | 4.31E+09 | 1.35E+08 | 80.941 |
| BAN_SH_BU-10_2016 | 25.380 | 3.68E+05 | 8.83E+09 | 3.92E+09 | 80.941 |
| BAN_SH_RN-21_2016 | 25.471 | 3.49E+05 | 8.37E+09 | 3.54E+09 | 80.941 |
| BAN_SH_RN-23_2016 | 29.179 | 4.67E+04 | 1.12E+09 | 1.84E+07 | 80.851 |
| BAN_SH_SN _26_2016 | 25.96 | 2.37 E+05 | 5.69 E+09 | 4.78E+08 | 81.253 |
| BAN_SH_SS_ 27_2016 | 32.728 | 2.22E+04 | 5.32 E+08 | 2.13E+07 | 81.253 |
| BAN_SH_SS_ 29_2016 | 30.767 | 6.41 E+04 | 1.54 E+09 | 1.23E+08 | 81.446 |
| BAN_SH_TA _33_2016 | 28.24 | 2.52 E+05 | 6.05 E+09 | 4.19E+08 | 81.253 |
| BAN_SH_MU-04_2016 | UND | - | - | - | 66.581 |
| BAN_SH_BU-11_2016 | UND | - | - | - | 66.562 |
| BAN_SH_BU-12_2016 | UND | - | - | - | 65.848 |
| BAN_SH_RN-24_2016 | UND | - | - | - | 67.366 |
| BAN_SH_RN-25_2016 | UND | - | - | - | 67.009 |
| BAN_SH_AS _11_2016 | UND | - | - | - | 65.569 |
| BAN_SH_AS _12_2016 | UND | - | - | - | 66.032 |
| BAN_SH_DE _01_2016 | UND | - | - | - | 67.167 |
| BAN_SH_SS _11_2016 | UND | - | - | - | 65.241 |
| BAN_SH_SS_ 12_2016 | UND | - | - | - | 66.691 |
| BAN_SH_TA _31_2016 | UND | - | - | - | 66.089 |
| BAN_SH_SU-1_2017 | 33.403 | 2.24E+05 | 5.38E+09 | 2.11E+08 | 80.941 |
| BAN_SH_SU-2_2017 | 30.043 | 1.30E+06 | 3.12E+10 | 3.60E+09 | 81.210 |
| BAN_SH_SU-3_2017 | 33.556 | 2.10E+05 | 5.04E+09 | 1.22E+09 | 81.446 |
| BAN_SH_SU-4_2017 | 33.2 | 2.50E+05 | 6.00E+09 | 1.30E+09 | 81.059 |
| BAN_SH_AL-1_2017 | 18.43 | 1.10E+10 | 2.64E+14 | 3.36E+13 | 80.941 |
| BAN_SH_AL-2_2017 | 27.563 | 5.20E+06 | 1.25E+11 | 2.11E+08 | 80.941 |
| BAN_SH_AL-3_2017 | 32.503 | 3.60E+05 | 8.64E+09 | 1.82E+09 | 81.446 |
| BAN_SH_AL-4_2017 | 24.28 | 9.37E+06 | 2.25E+11 | 8.87E+10 | 81.059 |
| BAN_SH_DE-1_2017 | 27.947 | 4.30E+06 | 1.03E+11 | 2.64E+09 | 80.866 |
| BAN_SH_DE-2_2017 | 29.744 | 1.60E+07 | 3.84E+11 | 3.36E+10 | 81.210 |
| BAN_SH_DE-3_2017 | 28.972 | 2.40E+06 | 5.76E+10 | 7.44E+09 | 81.446 |
| BAN_SH_KU-1_2017 | 27.748 | 4.80E+06 | 1.15E+11 | 3.36E+09 | 80.866 |
| BAN_SH_KU-2_2017 | 29.918 | 1.50E+06 | 3.60E+10 | 1.07E+10 | 80.866 |
| BAN_SH_KU-3_2017 | 30.315 | 1.19E+06 | 2.86E+10 | 1.37E+09 | 81.059 |
| BAN_SH_KU-4_2017 | 24.19 | 3.69E+07 | 8.86E+11 | 3.39E+10 | 81.446 |
| BAN_SH_SN-1_2017 | 33.749 | 1.87E+05 | 4.49E+09 | 6.24E+08 | 81.210 |
| BAN_SH_SN-2_2017 | 32.681 | 3.32E+05 | 7.97E+09 | 5.52E+08 | 81.210 |
| BAN_SH_SN-3_2017 | 24.17 | 2.01E+07 | 2.01E+07 | 6.79E+06 | 80.941 |
| BAN_SH_DE-4_2017 | UND | - | - | - | 65.848 |
| BAN_SH_SN-4 _2017 | UND | - | - | - | 67.366 |

**Table S2: Accession no., sequence IDs, sources and collection time of the WSSVs sequences of dataset.**

| **Accession no.** | **Sequence_ID** | **Source (Country)** | **Collection (Year)** |
| --- | --- | --- | --- |
| MF489075 | BAN_SH_SU-3_2015 | Bangladesh:Satkhira sadar, Satkhira | 04-Mar-15 |
| MF489076 | BAN_SH_DE-4_2015 | Bangladesh:Deabhata, Satkhira | 04-Mar-15 |
| MF489077 | BAN_SH_AL-2_2015 | Bangladesh:Assasuni, Satkhira | 04-Mar-15 |
| MF489078 | BAN_SH_SU-2_2017 | Bangladesh:Satkhira sadar, Satkhira | 12-May-17 |
| MF489079 | BAN_SH_SN-1_2017 | Bangladesh:Shymnagar,Satkhira | 12-May-17 |
| MF489080 | BAN_SH_KU-2_2017 | Bangladesh:Kaliganj,Satkhira | 12-May-17 |
| MF489081 | BAN_SH_DE-2_2017 | Bangladesh:Debhata,Satkhira | 12-May-17 |
| MF489082 | BAN_SH_AL-2_2017 | Bangladesh:Assasuni,Satkhira | 12-May-17 |
| KJ817413 | BAN SH AL-1 2014 | Bangladesh | 2014 |
| KJ817414) | BAN SH AL-2 2014 | Bangladesh | 2014 |
| KJ817415) | BAN SH DE-3 2014 | Bangladesh | 2014 |
| KJ817417 | BAN SH SN-7 2014 | Bangladesh | 2014 |
| KJ817418 | BAN SH RA-14 2014 | Bangladesh | 2014 |
| KJ817420 | BAN SH SN-16 2014 | Bangladesh | 2014 |
| KJ817416) | BAN SH SU-4 2014 | Bangladesh | 2014 |
| KJ817419 | BAN SH MU-15 2014 | Bangladesh | 2014 |
| KP219388 | BAN_SH_BG-1C_2014 | Bangladesh:Burigoalini, Satkhira | 2014 |
| KP219387 | BAN_SH_MU-23_2014 | Bangladesh | 2014 |
| KU556686.1 | VELLAR | India | 2015 |
| KT748521.1 | Mx12-1 | Mexico | 2012 |
| DQ681069.1 | Indian isolate | India | 2006 |
| AF502435.1 | China isolate | China | 2002 |
| DQ007315.1 | Xiang | China | 2005 |
| AY249440.1 | China-99/Qindao | China | 2003 |
| AY249443.1 | Japan-98 | Japan | 2003 |
| AY249442.1 | US-98/South | USA | 2003 |
| AY249441.1 | Indonesia-97 | Indonesia | 2003 |
| EU414753.1 | DDL2/2008 | India | 2008 |
| AY249434.1 | China-95/Dalian | China | 2003 |
| DQ979320.1 | China isolate | China | 2006 |
| HM484390.1 | Kadalur-NM4 | India | 2009 |
| HM484389.1 | Kadalur-NM3 | India | 2009 |
| HM484388.1 | Kadalur-NM2 | India | 2009 |
| HM484387.1 | Kadalur-NM1 | India | 2009 |
| HM484386.1 | Chidambaram-M8 | India | 2009 |
| HM484385.1 | Chidambaram-M7 | India | 2009 |
| HM484384.1 | Chidambaram-M6 | India | 2009 |
| HM484383.1 | Chidambaram-M5 | India | 2009 |
| HM484382.1 | Chidambaram-M4 | India | 2009 |
| HM484381.1 | Chidambaram-M3 | India | 2009 |
| AY324881.1 | Korea-01 | Korea | 2003 |
| AY682926.1 | China | China | 2006 |
| DQ013883.1 | Indian isolate | India | 2006 |
| DQ013882.1 | Indian isolate | India | 2005 |
| AY422228.1 | India isolate | India | 2003 |
| GQ328029.1 | Isolate-03 | Korea | 2009 |
| EU931451.2 | Mexico-Sinaloa | Mexico | 2008 |
| AF173993.1 | Netherlandian isolate | Netherlands | 1999 |
| FJ756456.1 | L1 | Mexico | 2001 |
| FJ756455.1 | WSSV-Mx-H-2004 | Mexico | 2004 |
| FJ756454.1 | WSSV-Mx-G-2004 | Mexico | 2004 |
| FJ756453.1 | WSSV-Mx-C-2005 | Mexico | 2005 |
| DQ098011.1 | China isolate | China | 2005 |
| KF723558.1 | Chabahar | Iran | 2011 |
| AF369029.2 | WSSV-TH | Thailand | 2001 |
| JX515788.1 | K-LV1 | SouthKorea | 2011 |
| EF534254.1 | Cochin | India | 2007 |
| AY873785.1 | SDDL18/04 | India | 2005 |
| AJ937859.1 | Mexican | Mexico | 2005 |
| JX027481.1 | KKD | India | 2012 |
| EF661844.1 | Indian isolate | India | 2007 |
| AY168644.1 | Vietnam | Vietnam | 2002 |
| AF380842.1 | SouthKorea | SouthKorea | 2001 |
| AB855742.1 | IRI-KHZ/904 | Iran | 2003 |
| AJ551447.1 | Vietnam | Vietnam | 2003 |
| JX444994.1 | 05VN.VP28.HCM2.12 | Vietnam | 2012 |
| JX444993.1 | 07VN.VP28.BD2.12 | Vietnam | 2012 |
| JX444992.1 | 06VN.VP28.BD1.11 | Vietnam | 2011 |
| HQ130032.1 | Brazil | Brazil | 2007 |
| EF194079.1 | Thailand | Thailand | 2006 |
| GU734034.1 | SDDL/09 | India | 2009 |
| DQ902658.1 | Indian isolate | India | 2006 |
| AF332093.3 | WSSV-CN | China | 2014 |
| NC003225.2 | CN01 | China | 2000 |
