# Supplementary figures and images for: Circulatory white spot syndrome virus in South-West region of Bangladesh from 2014 to 2017: molecular characterization and genetic variation
Source: AMB Express. 2018 Feb 20;8:25. doi: 10.1186/s13568-018-0553-z (PMC5818386; doi:10.1186/s13568-018-0553-z)

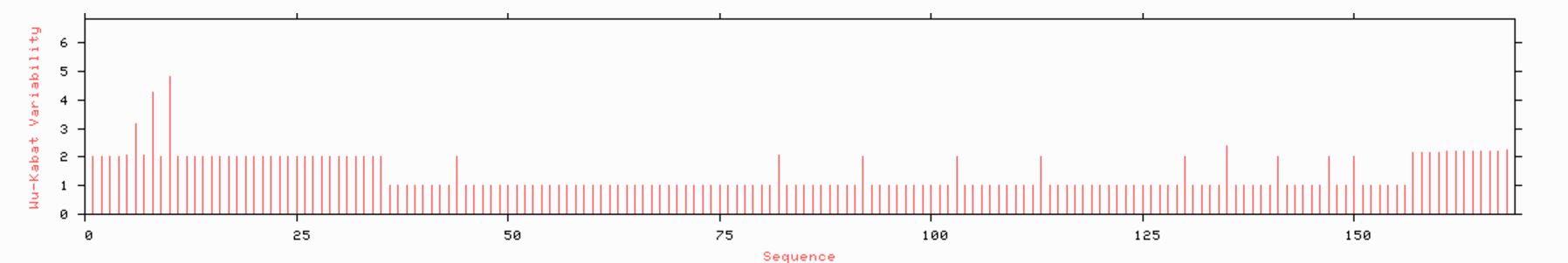

Supplement: Supplementary file 2 — Additional file 2: Figure S1. Wu-Kabat protein variability index showed 13 unique mutations as observed from the protruded peaks’ positioned along with the amino acid residue of VP28 protein. [file 13568_2018_553_MOESM2_ESM.jpg]

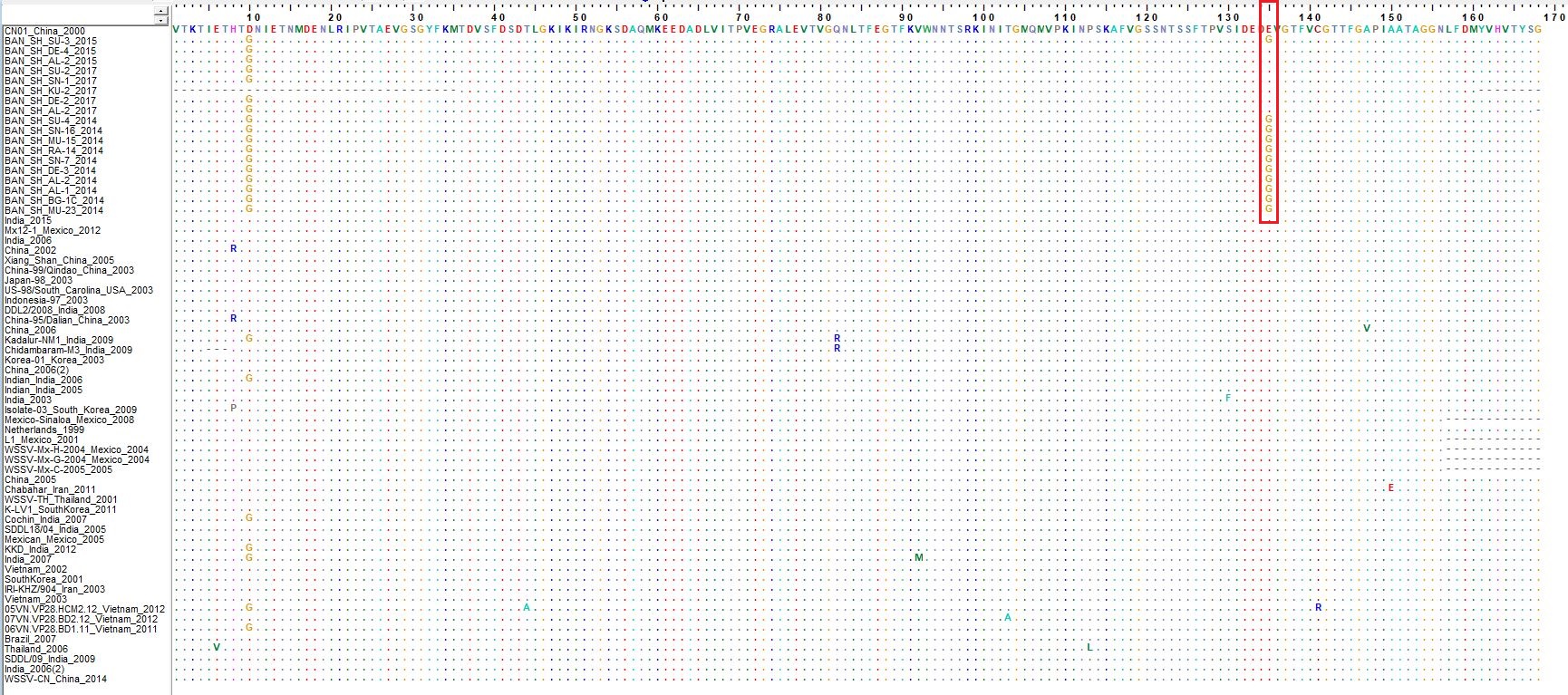

Supplement: Supplementary file 3 — Additional file 3: Figure S2. The aligned protein sequence data of VP28 taking into consideration 66 sequences generated by BioEdit Sequence Alignment Editor. The left portion of the frame contained the isolate ids of the sequences. The digit in the upper portion of the figure represented the residue number of protein. The red bar indicates the unique mutation position along with the mutated amino acid. [file 13568_2018_553_MOESM3_ESM.jpg]

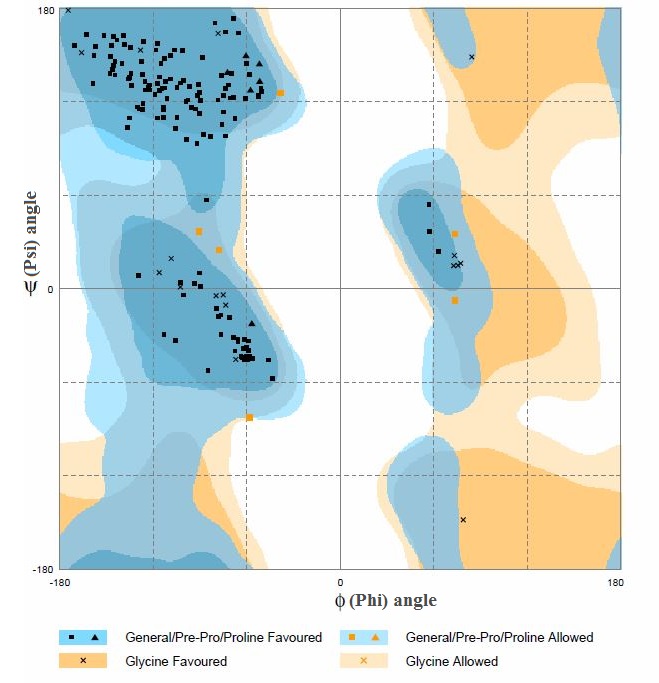

Supplement: Supplementary file 4 — Additional file 4: Figure S3. Ramachandran plot of modeled VP28 protein of WSSV. The number of residues in favored, allowed and outlier region are 96.4, 3.6 and 0%, respectively. On the X and Y axes, ϕ (phi) and ψ (psi) represent the torsion angles around Cα (alpha carbon) to amine and carboxyl group of different amino acids. [file 13568_2018_553_MOESM4_ESM.jpg]
